# Epigenetic Approaches to the Treatment of Dental Pulp Inflammation and Repair: Opportunities and Obstacles

**DOI:** 10.3389/fgene.2018.00311

**Published:** 2018-08-07

**Authors:** Michaela Kearney, Paul R. Cooper, Anthony J. Smith, Henry F. Duncan

**Affiliations:** ^1^Division of Restorative Dentistry & Periodontology, Dublin Dental University Hospital, Trinity College Dublin, University of Dublin, Dublin, Ireland; ^2^Oral Biology, School of Dentistry, College of Medical and Dental Sciences, University of Birmingham, Birmingham, United Kingdom

**Keywords:** tertiary dentinogenesis, caries, non-coding RNA, dental pulp, mesenchymal stem cells, regenerative endodontics, epigenetics, histone deacetylase inhibitors

## Abstract

Concerns over the cost and destructive nature of dental treatment have led to the call for novel minimally invasive, biologically based restorative solutions. For patients with toothache, this has resulted in a shift from invasive root-canal-treatment (RCT) toward more conservative vital-pulp-treatment (VPT) procedures, aimed to protect the pulp and harness its natural regenerative capacity. If the dental pulp is exposed, as long as the infection and inflammation can be controlled, conservative therapies can promote the formation of new tertiary dentine in a stem cell-led reparative process. Crucially, the volume and quality of new dentine is dependent on the material applied; however, currently available dental-materials are limited by non-specific action, cytotoxicity and poor clinical handling. Looking to the future, an improved understanding of the cellular regulators of pulpal inflammation and associated repair mechanisms is critical to predict pulpal responses and devise novel treatment strategies. Epigenetic modifications of DNA-associated proteins and the influences of non-coding RNAs have been demonstrated to control the self-renewal of stem cell populations as well as regulate mineralised tissue development and repair. Notably, the stability of microRNAs and their relative ease of sampling from pulpal blood highlight their potential for application as diagnostic inflammatory biomarkers, while increased understanding of their actions will not only enhance our knowledge of pulpal disease and repair, but also identify novel molecular targets. The potential therapeutic application of epigenetic modifying agents, DNA-methyltransferase-inhibitors (DNMTi) and histone-deacetylase-inhibitors (HDACi), have been shown to promote mineralisation and repair processes in dental-pulp-cell (DPC) populations as well as induce the release of bioactive dentine-matrix-components. Consequently, HDACis and DNMTis have the potential to enhance tertiary dentinogenesis by influencing the cellular and tissue processes at low concentrations with minimal side effects, providing an opportunity to develop a topically placed, inexpensive bio-inductive restorative material. The aim of this review is to highlight the potential role of epigenetic approaches in the treatment of the damaged dental pulp, considering the opportunities and obstacles, such as off-target effects, delivery mechanisms, for the therapeutic use of miRNA as an inflammatory biomarker or molecular target, before discussing the application of HDACi and DNMTi to the damaged pulp to stimulate repair.

## Introduction

Inflammation of the dental pulp (pulpitis) generally presents with severe pain as toothache, which is commonly treated by either extracting the tooth or root canal treatment (RCT). Dental decay (caries) and microleakage around dental restorations remain the most common causes of pulpitis and highlight the essential role of microbial infection in the disease (Kakehashi et al., 1965; Brännström and Nyborg, 1973; Sengupta et al., 2017). If the microbial stimulus is untreated, it will induce progressive inflammatory reactions in the pulp, leading to pain, tissue necrosis and abscess formation (Farges et al., 2015); however, if the stimulus is removed prior to pulp necrosis and the tooth is adequately restored, then resolution of pulpitis and tissue repair is possible (Tronstad and Mjör, 1972).

Recent concerns over the cost and destructive nature of dental treatment have led the profession to explore novel methodologies that may develop regenerative treatments and promote minimally invasive, biologically based dental restorative solutions (Schmalz and Smith, 2014). For patients with pulpitis and toothache, this has resulted in a shift from RCT toward more conservative dental procedures aimed at protecting the pulp and harnessing its natural regenerative capacity (Ferracane et al., 2010). If the nerve (or pulp) of the tooth is exposed by decay, conservative therapies can “wall off” the pulp by promoting the formation of new dentine in a stem cell (SC) led reparative process (Smith et al., 1995a,b), thus preventing the need for pulpectomy and RCT. Crucially, the volume and quality of the repair is dependent on the dental material applied (Nair et al., 2008); however, currently many of the available dental materials are limited by their cytotoxicity, non-specific action, poor handling and unpredictable reparative capacity (Duncan et al., 2011).

Traditionally, vital pulp treatment (VPT) procedures have been unpredictable due in part to the diagnostic difficulty of accurately assessing the inflammatory state of the pulp clinically (Barthel et al., 2000; Ricucci et al., 2014). Next generation diagnostics are being developed, designed to discriminately assess the inflammatory state of the pulp at chairside using blood or another tissue fluid to assess quantitatively biomarkers of disease (Mente et al., 2016). At present, several inflammatory cytokines and tissue proteases have been preliminarily investigated, but identifying a reliable, stable indicator of pulpitis has thus far not been achieved (Rechenberg et al., 2016). Non-coding RNAs and specifically microRNAs (miRNA) have significant potential in this regard, as they are detectable and stable in body fluids and have been successfully employed to discriminately assess inflammation at other sites of the body (Schönauen et al., 2018).

Exciting opportunities also exist for development of new dental biomaterials targeted at pulp regenerative processes, which avoid the need for destructive RCT during toothache. A potential novel solution is to target the process of DNA-associated histone acetylation, which is balanced by histone-acetyltransferases (HAT) and histone-deacetylases (HDAC), as we have shown that altering this balance increases dental pulp cell (DPC) mineralisation and promotes reparative processes within the damaged pulp (Duncan et al., 2013, 2016b). The epigenetic modifying agents, histone deacetylase inhibitors (HDACis), can therapeutically alter this balance leading to the accumulation of acetylated proteins, with transcriptional and cellular effects. Although HDACis, such as suberoylanilide hydroxamic acid (SAHA) and panobinostat, are used therapeutically in cancer (Bolden et al., 2006), their therapeutic application is also being investigated in osteogenesis (Boer et al., 2006) and inflammatory diseases (Leoni et al., 2002); however, their use in dentistry would be novel (Duncan et al., 2011).

At present, significant *in vitro* research into the use of therapeutic epigenetic modification has highlighted focus areas for further translational investigation. This will involve overcoming the challenges of sustained, controlled topical delivery of the pharmacological inhibitor as well as regulatory and ethical considerations. The aim of the current review is to analyse the methods currently used to diagnose and treat dental pulp disease, identifying their deficiencies, while providing an in-depth assessment of the potential role for the clinical use of epigenetic modification in improving the clinical treatment strategies for dental pulp inflammation and repair.

## Review

### Dental Pulp Disease and the Limitations of Current Therapies

The dental pulp occupies the centre of the tooth being encased in health by an outer layer of dentine and enamel. The odontoblast is responsible for dentine formation during development and is located at the periphery of the pulp in contact with dentine, forming a functionally linked tissue known as the pulp-dentine complex (Pashley, 1996). After the completion of tooth development (primary dentinogenesis), the pulp is not a redundant tissue, as it continues to lay down secondary dentine throughout the life of the tooth, acts as a sensor for microbial insult, pain and temperature and mediates the formation of tertiary dentine in response to irritation or disease (Simon et al., 2009). There are two types of tertiary dentine formed depending on the severity of the irritating stimulus; mild irritation induces an up-regulation of existing odontoblast activity to form reactionary dentine, while stronger stimuli result in odontoblast death and the recruitment of dental pulp stem/progenitor cells, which differentiate into odontoblast-like cells under the influence of bioactive molecules to form reparative dentine (Lesot et al., 1994).

The pulp can be irritated by several external stimuli including caries, trauma and as a result of restorative dental procedures, all of which stimulate inflammatory responses in the pulp, with the nature and extent of the pulpitis reflecting the severity of the challenge (Mjör and Tronstad, 1972). Dental caries (tooth decay) is the primary cause of pulpitis and remains one of the most prevalent infectious diseases worldwide, with recent reports indicating that over 90% of adults have experienced the disease (Dye et al., 2015). Microbial infection challenges the pulp as bacterial products diffuse through the dentinal tubules, inducing inflammation even when the carious process or restoration has not yet reached the pulp (Warfvinge and Bergenholtz, 1986). As the carious process advances toward the pulp tissue, the inflammation intensifies and the nature of the bacterial microflora alters to the predominately anaerobic flora identified in deep carious lesions (Nadkarni et al., 2004; Chhour et al., 2005). If the carious infection progresses unchecked, the microbial biofilm will advance toward the pulp and the associated bacteria will invade the tissue; this aggressive bacterial challenge invariably leads to irreversible Pulpitis, pulp necrosis and subsequent apical periodontitis (Reeves and Stanley, 1966). Pulp necrosis will necessitate urgent remedial dental treatment, such as tooth extraction or RCT to preserve the tooth, which are destructive and can lead to considerable patient expense as well as weakening of the residual tooth structure (Yang et al., 2016). In addition, although RCT is a common dental procedure it is not always effective with reports of long-term failure in 10–50% of cases dependent on the skill of the operator (Di Filippo et al., 2014), efficacy of chemo-mechanical debridement and the patients innate response to treatment (Ng et al., 2011).

As pulp tissue has an innate ability to heal if the challenge is removed and the tooth is suitably restored (Mjör and Tronstad, 1974), recent attention has focused on the development of biologically based minimally invasive biomaterial solutions, which maintain the vitality of the pulp (Taha and Abdelkhader, 2018). Forming part of a regenerative endodontic approach, vital pulp treatments aim to induce the natural regenerative ability of the dental pulp in order to repair the tooth, while maintaining pulp tissue that would have been removed in traditional therapies (Murray et al., 2007). Traditionally, vital pulp treatment has demonstrated poor outcomes (Barthel et al., 2000), at least in part due to a poor understanding of the pulpal reparative processes, inadequate dental restorative materials and lack of sensitive diagnostic tools capable of accurately determining the inflammatory state of the tissue (Nair et al., 2008; Rechenberg et al., 2016). Traditional vital pulp materials, including calcium hydroxide (Ca[OH]_2_), have demonstrated limited clinical success in treating pulpitis, in part due to their inability to prevent microleakage at the tooth-material interface (Bergenholtz et al., 1982; Hilton, 2002) as well as the toxicity of their constituents (Mantellini et al., 2003). The advent of new tricalcium silicate materials, such as mineral trioxide aggregate (MTA), has offered improvements over existing materials, demonstrating superior histological responses (Nair et al., 2008), as well as improved clinical outcomes (Mente et al., 2014).

Current VPT materials, like Ca[OH]_2_ and MTA, stimulate a reparative response by non-specific mechanisms (Sangwan et al., 2013), which is likely to involve a modulation of pulpal inflammation and creation of an environment conducive to repair, which likely involves local release of tissue-bound growth factors and cytokines (Graham et al., 2006; Tomson et al., 2007). The non-specific nature of these materials’ actions creates problems for scientists and clinicians in elucidating both the specific mechanistic response to material application and also in devising targeted-repair solutions. Ca[OH]_2_ also exhibits other limitations, as the reparative hard tissue barrier formed beneath these materials is generally incomplete, with the resulting defects often associated with pulpal inflammation or necrosis (Cox et al., 1996). Although MTA induces more complete hard tissue barriers (Nair et al., 2008), limitations with regards to handling concerns and post-operative tooth discolouration have become evident (Felman and Parashos, 2013).

Although it is clear that uncontrolled pulpitis will inhibit reparative events, it also appears that a low level of inflammation is critical, at least initially, to promote reparative processes (Cooper et al., 2010). Therefore, creating a controlled environment in the pulp in which we can accurately predict the level of inflammation, while applying next generation smart biomaterials to target repair processes appears central to the development of more predictable VPT solutions.

### Epigenetics and Epigenetic Modulating Agents

Epigenetic modifications play an essential role in cell development and differentiation by regulating gene expression without altering the DNA sequence (Allis and Jenuwein, 2016). Recently, the importance of epigenetic influence on the regulation of embryonic stem cell (ESC) and dental pulp stem cell (DPSC) fate, as well as the therapeutic potential of controlling DPSC self-renewal and differentiation has been highlighted (Bayarsaihan, 2016; Duncan et al., 2016a; Rodas-Junco et al., 2017). Several epigenetic-regulatory mechanisms have been described, with DNA methylation and histone modification being the most thoroughly investigated. Over the last few years, the emerging role of non-coding RNAs (ncRNAs) in the epigenetic regulation of gene expression has been the subject of intensive research (Peschansky and Wahlestedt, 2014). As these transcriptional mechanisms are implicated in the maintenance of health and the biological response to disease, including inflammatory and reparative processes, there is an opportunity to target epigenetic modifications as diagnostic biomarkers or as part of a dental therapeutic strategy.

#### DNA Methylation

DNA methylation involves the transfer of a methyl group to a cytosine base of DNA, converting it to 5-methylcytosine (Bird, 2002). In mammals, this process is controlled by four DNA methyltransferase enzymes (DNMTs), which invariably modify the cytosine residue in a cytosine-guanine (CpG) dinucleotide. Three of the DNMTs, -1, -3A, and -3B, are involved in the generation and maintenance of DNA methylation in cells, while DNMT-3L has no enzymatic activity (Kareta et al., 2006). DNA methylation results in gene repression, generally by either physical prevention of DNA-binding proteins (e.g., transcription factors) associating with their target site or by the methylated CpG dinucleotide attracting methyl-CpG-binding proteins (MBDs), which subsequently attract co-factors associated with transcriptional repression such as chromatin remodellers, histone deacetylases and histone methyltransferases (Bird and Wolffe, 1999). DNA methylation is considered a ‘stable’ epigenetic mark, with methylation patterns retained throughout cell division, providing a form of cellular memory (Kim and Costello, 2017).

DNA methylation plays a critical role in the regulation of gene expression, with inherited disorders such as Angelman syndrome and Prader–Willi syndrome demonstrating the effect of aberrant DNA methylation (Horsthemke and Wagstaff, 2008). The de-regulation of DNA methylation in cancer has identified DNA methylation as a key therapeutic target when developing new chemotherapeutic drugs. The currently available pharmacological DNMT inhibitor (DNMTi), 5-azacytidine, functions by incorporating into the DNA structure, thereby preventing the interaction between DNA and DNMTs and stimulating DNMT degradation. 5-azacytidine has FDA approval to treat patients suffering from myelodysplastic syndromes (MDS) (Yang et al., 2010).

#### Histone Modifications

In eukaryotes, chromatin consists of DNA tightly wrapped around an assortment of histone proteins in order to compress 2 metres of DNA into a nucleus approximately 6 μm wide. The basic repeating unit of chromatin is the nucleosome, which represents 8 histone proteins and an associated 146 base pairs of DNA (Luger et al., 1997). Histone proteins are subject to a number of post-translational modifications (PTMs), such as acetylation, methylation, phosphorylation and sumoylation. These modifications principally occur on the N-terminal, which protrudes from the nucleosome core as a ‘tail’, and result in the modulation of gene expression (Jenuwein and Allis, 2001). The acetylation process is balanced by histone acetyltransferases (HATs), which add a negatively charged acetyl group and weaken the interaction between DNA and histone residues, and histone deacetylases (HDACs), which remove the acetyl group. There are four classes of HDACs, with the zinc-dependent classes I, II and IV being referred to as the ‘classical’ HDACs, while class III, the sirtuins, are dependent on NAD^+^ (Seto and Yoshida, 2014). Increased HAT activity creates an open chromatin architecture readily accessible by transcriptional machinery (Hebbes et al., 1988), while HDAC activity generally condenses the chromatin structure making it transcriptionally repressive (Eberharter and Becker, 2002). Similarly, histone methylation is mediated by histone methyltransferases (HMTs) and can facilitate or impede gene expression depending on which residue is methylated and the number of methyl groups added (Barski et al., 2007). For example, trimethylation of lysine 27 on histone 3 (H3K27me3) is correlated with gene silencing, while trimethylation of lysine 4 on histone 3 (H3K4me3) is associated with active transcription of nearby genes (Cao et al., 2002; Guenther et al., 2007). Although there is a didactic tendency to consider HATs, HDACs and HMTs in isolation, in reality a complex interplay occurs between modifications, which will shape the epigenetic landscape (Fischle et al., 2003).

The dysregulation of HATs and HDACs has an important role in human disease, highlighted by the involvement of the HAT transcriptional cofactor CREB-binding protein (CBP) in the pathogenesis of Huntington’s disease (HD) (Nucifora et al., 2001; Steffan et al., 2001). The mutant *huntington* gene in HD patients sequesters CBP, the loss of which results in hypo-acetylation of histone proteins in neurons and subsequent deregulation of neuronal gene expression (Ryu et al., 2006; Sadri-Vakili et al., 2007; McFarland et al., 2012). In addition, asthma sufferers have increased HAT and decreased HDAC activity, resulting in increased inflammatory gene expression (Ito et al., 2002). The corticosteroids used to treat asthma exert at least part of their anti-inflammatory effects by recruiting HDAC-2 to activated inflammatory genes (Barnes et al., 2005).

The association of HDACs and HMTs with human disease has generated an interest in the development of HDAC- and recently HMT-based therapeutics. HDACis in particular have shown potential for use in treatment of cancer (Wagner et al., 2010), neurodegenerative disorders (Chuang et al., 2009) and inflammatory diseases (Adcock, 2007).

#### Non-coding RNA

Gene expression can also be epigenetically regulated through the activity of non-coding RNA (ncRNA), which includes small interfering RNA (siRNA), miRNA and long ncRNA (lncRNA), with the number of ncRNA far outnumbering coding mRNA transcripts (Palazzo and Lee, 2015). Both siRNAs and miRNAs mediate gene silencing by being incorporated into the RNA-induced silencing complex (RISC) and guiding it to a target mRNA strand. The target mRNA is then cleaved by Argonaute 2 (AGO2)-mediated cleavage. Both miRNA and siRNA differ in their specificity, with the former mediating degradation of multiple targets, while the latter has only one target (Carthew and Sontheimer, 2009). The extent of miRNA-mediated gene regulation is continually evolving, but at least one-third of the genome is estimated to be regulated by miRNAs (Lewis et al., 2005). The complexity of these regulatory mechanisms is amplified by the fact that more than one miRNA can act on a single gene, and a single miRNA can act on many targets. This intricate network of miRNAs and their target genes is an integral component of the mechanisms regulating cellular processes in health, with miRNAs often being deregulated in human diseases, including inflammatory diseases such as rheumatoid arthritis (Nakasa et al., 2008) and multiple sclerosis (de Faria et al., 2012). This deregulation, coupled with their high stability in body fluids and ease of quantification, makes them promising candidates for use as diagnostic biomarkers in these conditions (Mi et al., 2013). Notably, the mechanism by which miRNAs mediate targeted-gene silencing also provides an opportunity to develop miRNA-based therapeutics in dental disease, with the potential to silence aberrantly overexpressed genes. Indeed, several miRNA-based therapeutic strategies have already been developed and are undergoing pre-clinical and clinical trials in the treatment of diseases such as Hepatitis C (Janssen et al., 2013; van der Ree et al., 2014) and cancer (Beg et al., 2017).

Long non-coding RNAs (> 200 nucleotides) constitute the largest family of ncRNA transcripts in the human genome. Their exact role in gene expression remains unclear; however, they are known to participate in gene regulation at the transcriptional, post-transcriptional and epigenetic level (Kung et al., 2013). The diagnostic benefits of the upregulated lncRNA *HULC* in hepatocellular carcinoma (HCC) have been reported (Panzitt et al., 2007) as has the potential therapeutic inhibition of lncRNA in the prevention of HCC progression, with knockdown of lncRNA 00974 in a HCC cell-line stimulating cell cycle arrest, while inhibiting cell proliferation and invasion *in vitro* (Tang et al., 2014).

In summary it is evident that epigenetic mechanisms play a vital role in the orchestration and regulation of gene expression, thereby offering the translational potential of therapeutic intervention in many medical fields. The remainder of this review will focus on the use of epigenetic therapeutics within inflammation and repair specifically in relation to the tooth and the dental pulp.

### DNA Methylation in the Dental Pulp: Inflammation, Regeneration and Repair

DNA methylation is crucial to establishing and stabilising cellular identity during differentiation. After a cell has committed to a particular fate, a specific pattern of gene expression is established, with DNA methylation central in the repression of non-essential genes. The stability of methylation prevents aberrant differentiation of daughter cells, which could disrupt tissue development (Hemberger et al., 2009), and is evident with induced pluripotent SCs (iPSCs) retaining a DNA methylation pattern characteristic of the somatic tissue from which they were derived, and indeed, following the same lineage as the donor cells (Kim et al., 2010).

#### General Studies

DNA methylation plays a prominent role in reparative and regenerative processes. For example, when retinal neurons in zebrafish are damaged, locally derived Müller glial cells dedifferentiate into multipotent progenitors, re-enter the cell cycle and differentiate into new retinal neurons (Powell et al., 2013). The reprogramming is initially accompanied by demethylation of a small portion of the Müller glial genome, followed by an increase in methylation during differentiation and migration of the retinal progenitors, a process which can be blocked by the DNMTi 5-aza-2′-deoxycytidine (Powell et al., 2013). This highlights a likely role for DNA methylation/demethylation in this cellular repair process. DNA methylation also contributes to inflammatory processes, with hypomethylation of the promoter site of the Toll-like receptor-2 (*TLR2*) gene being associated with a pro-inflammatory response to bacterial infection in bronchial epithelial cells (Shuto et al., 2006). *TLR4* has also been shown to be regulated by a combination of DNA methylation and histone acetylation in intestinal epithelial cells, contributing to the maintenance of intestinal homeostasis (Takahashi et al., 2009).

#### Dental Studies

Advances in our general understanding of the role of DNA methylation in repair and inflammation have been translated to the injured and damaged dental pulp. Altered methylation status of inflammation-associated genes has been reported, with the interferon gamma (*IFN*-γ) gene only partially methylated or unmethylated in 93% of inflamed pulp tissue samples, compared with normal pulp tissues of which 44% exhibited total methylation. Furthermore, the pulp tissues demonstrating total methylation had no *IFN*-γ transcription (Cardoso et al., 2010). Recently, ten-eleven translocation 2 (TET2), a methylcytosine dioxygenase, which promotes DNA demethylation by converting 5-methylcytosine to 5-hydroxymethylcytosine, was demonstrated to play a role in the regulation of dental pulp inflammation. In an *in vitro* culture model, TET2 enhanced lipopolysaccharide (LPS)-induced inflammation in human via the regulation of MyD88 hydroxymethylation, highlighting the increasing importance of epigenetic regulation in dental pulp inflammation (Wang et al., 2018). Currently, there is a paucity of investigations into the epigenetic influence during dental pulp inflammation and therefore it may be some time before the precise relationship between these processes is elucidated. It has been highlighted that TLR2 and TLR4 are expressed at a higher level in inflamed pulp than in normal pulp; however, when the methylation patterns of *TLR2* and *CD14* (TLR4 co-receptor) were analysed, they were found to be similarly hypomethylated in both normal and inflamed pulp tissue (Mutoh et al., 2007; Cardoso et al., 2014). This highlights an unusual hypomethylation of these genes in the dental pulp and the need for further research into the relationship between TLR promoter methylation and dental pulp inflammation. Differential methylation patterns have also been highlighted in neighbouring inflamed periodontal tissues, with the promoters of the cytokine IFN-γ being hypomethylated, and the resulting cellular level of IFN-γ being elevated in comparison with normal periodontal tissues (Zhang et al., 2010).

As the accurate diagnosis of pulpal inflammation has long been problematic in dentistry (Ricucci et al., 2014), the observation that methylation patterns differ between healthy and inflamed pulps points to a potential therapeutic opportunity for employing differentially methylated genes as biomarkers for diagnosing pulpal disease. A recent study investigating the epigenetic regulation of inflammation in periodontal tissues found that the activation of the pro-inflammatory nuclear factor kappa-light-chain-enhancer of activated B cells (NF-κB) relied upon the methylation status of Smad6, which in its methylated form inhibits NF-κB activation (Zhang et al., 2018). Although this is a case of protein methylation rather than DNA methylation, this research highlights a potential opportunity to manipulate the methylation status of Smad6 in order to regulate the patient’s inflammatory response. Furthermore, methylation-based therapies could be developed in order improve the predictability of treating pulpitis. Only one study has investigated the reparative mineralisation effects of 5-aza-2′-deoxycytidine on dental pulp cells (Zhang et al., 2015) and demonstrated that although cellular proliferation was decreased in the presence of the DNMTi, mineralisation- and dental-associated genes, including dentin-sialophosphoprotein (*DSPP*), dentine-matrix-protein-1 (*DMP-1*), osterix (*Ox*) and *RUNX2*, were all upregulated and calcific nodule formation was accelerated (**Table 1**). Although only an *in vitro* study, it was concluded that DNMTi application could offer new therapeutic avenues for the damaged dental pulp (Zhang et al., 2015).

**Table 1:** Table presenting various studies which have investigated the effects of histone deacetylase inhibitors (HDACis) and DNA methyltransferase inhibitors (DNMTis) on mineralisation and differentiation in dental pulp cell populations.

Reference	Cell population	HDACi(s)	Mineralisation-associated gene expression changes
Duncan et al., 2012	DPC-line (MDPC-23)	TSA, VPA	**Up:** *BMP-4, DMP-1*, *TGF*-β-*1*
Kwon et al., 2012	DPC-line (MDPC-23)	SAHA	**Up:** *DMP-1, DSPP, ALP, Nestin, Nfic*
Duncan et al., 2013	Rat primary DPC	TSA, VPA	**Up:** *BMP-2, -4, DMP-1, DSPP, Nestin*
Jin et al., 2013	Human DPSCs	TSA	**Up**: *BSP, DMP-1, DSPP*
			**Down:** *OC*
Paino et al., 2014	Human DPSCs	VPA	**Up:** *BSP, OPN*
			**Down:** *OC*
Duncan et al., 2016b	Rat primary DPC	SAHA	**Up:** MMPs and endochondral ossification pathway
Liu et al., 2018	Human DPSCs	LMK-235	**Down:** Cell cycle, DNA replication pathways
			**Up:** *DSPP, ALP, RUNX2*
**Reference**	**Cell population**	**DNMTi(s)**	**Mineralisation-associated Gene Expression Changes**
Zhang et al., 2015	Human DPSCs	5-aza-2′-deoxycytidine	**Up**: *DSPP, DMP-1, OSX, RUNX2, DLX5, ALP*


*ALP, alkaline phosphatase; BMP-2, bone morphogenetic protein 2; BMP-4, bone morphogenetic protein 4; DSPP, dentin sialophosphoprotein; BSP bone sialoprotein; DMP-1, dentin matrix protein 1; Nfic, nuclear factor I/C; OC, osteocalcin; OPN, osteopontin; RUNX2, runt-related transcription factor 2; TGF-β-1, transforming growth factor beta-1.*

The research into the role of DNA methylation in regulating dental pulp inflammation and repair is preliminary in nature but offers promise. Future investigations should focus on *in vivo* experimentation of DNMTi application to the tooth as well as an assessment of the possibilities of utilising chairside assays measuring DNA methylation to assess the severity of pulpal inflammation.

### Histone Modifications and the Dental Pulp: Development and Differentiation

The critical nature of post-translational histone modifications in development and differentiation is evident when the *Ezh2* gene, mediating histone methylation, is deleted; this leads to death in early developmental stages in mice (O’Carroll et al., 2001). Abolition of acetylation processes in mice via HDAC-1 gene deletion also results in early embryonic lethality (Lagger et al., 2002). Furthermore, HDAC-1-deficient mouse ESCs preferentially differentiate into mesodermal cells such as cardiomyocytes, compared with HDAC-2-deficient ESCs, which demonstrate normal differentiation properties (Dovey et al., 2010). Treatment of ESCs with an HDACi, trichostatin A (TSA), results in a morphological and molecular phenotype characteristic of early differentiation stages. This phenotype closely mimics the early differentiation induced by removal of leukaemia inhibitory factor (LIF) from the cell culture, which is primarily used to maintain ESCs in their undifferentiated, pluripotent state (McCool et al., 2007).

Given the evidently pivotal role of histone modifications in development and differentiation, it is no surprise that severe disorders arise when these modifications are aberrantly distributed (Haberland et al., 2009). Thus, a comprehensive understanding of the individual histone modifications and their role in health, development and regeneration are vital when designing therapeutic solutions using inhibitors.

#### General Studies

Epigenetic modifications present an attractive therapeutic focus due to their association with disease and because they are relatively easy to reverse pharmacologically (Kelly et al., 2010). Indeed, epigenetic-modifying agents targeting histone acetylation have been shown to be effective in reducing inflammation, promoting mineralisation and modulating regenerative processes in a range of cell types (Shanmugam and Sethi, 2013; Gordon et al., 2015). For example, TSA has been shown to ameliorate symptoms of experimental autoimmune encephalomyelitis (EAE), a murine model of multiple sclerosis (MS). The potential uses of HDACis have also been investigated in the treatment of asthma (Ren et al., 2016), arthritis (Angiolilli et al., 2017) and diabetes (Larsen et al., 2007). Studies investigating the potentially regenerative effects of HDACi have indicated an increase in SC markers following digit amputation in mice (Wang G. et al., 2010) and during kidney organogenesis in zebrafish (de Groh et al., 2010) after HDACi administration.

#### Dental Studies

Within dental pulp research, histone acetylation and methylation have been shown to be involved in the development and repair of dentine. *DSPP* and *DMP-1*, both mineralisation markers associated with odontoblast activity, were found to be repressed by H3K27me3 in dental follicle progenitor cells (DFPCs), which give rise to the unmineralised dental follicle and periodontal ligament (Gopinathan et al., 2013). This was in contrast with DPSCs, in which the repressive H3K27me3 mark was almost absent. As a result, mineralising DPSCs had higher levels of *DSPP* and *DMP-1*, consistent with their eventual differentiation into pulp and dentine tissue (Gopinathan et al., 2013). Furthermore, class I and II HDACs are differentially distributed throughout dental pulp, with HDAC-2 and HDAC-9 being expressed in several pulpal cell populations and strongly expressed in mature odontoblasts, while HDAC-1, HDAC-3, and HDAC-4 only appear at low levels within the pulp, demonstrating tissue and time-specific expression of HDACs, suggesting distinct roles for each enzyme during dentine formation and repair (Klinz et al., 2012). As a result, the therapeutic development of the pan- or isoform-specific inhibition of HDAC has been the most actively researched target within the range of histone modifications.

#### HDAC Inhibitors

As the upregulation of HDAC activity is commonly associated with human disease, the development of HDACis has been the focus of intensive research in recent years. Indeed, HDACis have already been investigated for use in treating cancer (Lakshmaiah et al., 2014), inflammatory disease (Hull et al., 2016), neurodegenerative disorders (Didonna and Opal, 2015) and HIV/AIDS (Delagrèverie et al., 2016). There are currently 18 known human HDACs, divided into 4 classes based on the homology of their accessory domains to yeast HDACs. Zinc-dependent HDACs belong to classes I, II and IV, and share a high sequence conservation (de Ruijter et al., 2003). For this reason, most HDACis, in general and within dentine-pulp research, are pan-HDACis, targeting HDACs in classes I, II and IV with little discrimination (**Table 1**).

#### HDACis in Regenerative Endodontics

**FIGURE 1:**
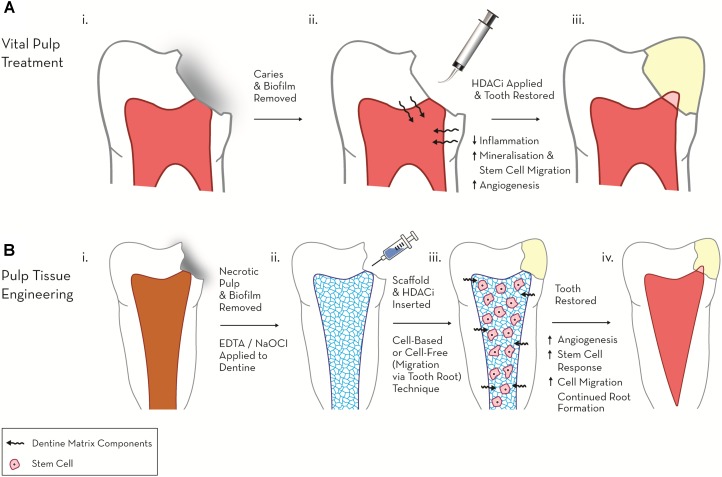
Schematic diagram highlighting the therapeutic potential of HDACi in regenerative endodontics. **(A)** Vital pulp treatment. (i) Deep carious lesion exposes pulp tissue. (ii) HDACi topically applied to exposed pulp, potentially as a component of a dental restorative material [e.g., mineral trioxide aggregate (MTA), calcium hydroxide, resin-based composite (RBC)] promotes tissue-repair processes (mineralisation, modulated inflammation and cell migration). (iii) Tooth permanently restored with amalgam and mineralised bridge formation evident under dental pulp capping material. **(B)** Pulp tissue engineering. (i) Pulp necrosis (ii) Necrotic tooth chemo-mechanical debrided and EDTA, NaOCl applied to dentine to release matrix components. (iii) HDACi applied within a cell or non-cell based scaffold stimulates further growth factor release, cell migration and differentiation. (iv) Tooth restored and new pulp-like tissue formed promoting continued root growth and restoring tooth tissue vitality.

Several HDACis have shown considerable promise in the field of dental pulp regeneration, in particular these include the pan-HDACis, TSA, SAHA and valproic acid (VPA) (**Table 1**). In a series of recent studies these HDACis have been shown to stimulate differentiation and promote migration in DPC cultures, suggesting a potential role in the treatment of the exposed dental pulp (Duncan et al., 2012; Jin et al., 2013; Paino et al., 2014). In the first of these pulpal studies, two HDACis (TSA, VPA), reduced proliferation and increased mineralisation dose-dependently in a dental-papillae derived cell line, while the application of HDACi in a range of relatively low concentrations only adversely influenced cell viability and cell cycle at the highest of the concentrations employed (Duncan et al., 2012). This work introduced the principle of HDACi-induced pulp cell mineralisation, but generated further questions regarding the potential nature of the response in primary/non-transformed cell cultures, which have been demonstrated to react differently to HDACi in other cellular systems (Ungerstedt et al., 2005; Lee et al., 2010). Subsequent primary DPC experimentation highlighted that HDACi induced reparative-related cellular responses at concentrations which did not stimulate significant cytotoxic effects; however, there were marked differences between the primary DPC and immortalised cell-line responses, which indicated a resistance of DPCs to HDACi-mediated toxicity (Duncan et al., 2013). Notably, TSA and VPA induced mineralisation at concentrations 10-fold lower in primary DPCs compared with the pulp cell-line. In relation to HDACi dosage, an increase in mineralisation was evident after an initial (24 h) application of HDACi in the primary DPC group, but not after constant HDACi dosage (14 days), which conversely resulted in an inhibition of differentiation (Duncan et al., 2013). This effect was not evident in the cell line cultures in which constant exposure to HDACi increased mineralisation. From a translational perspective, the stimulation of mineralisation in DPCs without toxicity after a short-term dose of HDACi supported the potential chairside application of these materials as pulp capping agents within vital pulp treatment (**Figure 1**).

A further study analysed the effect of TSA on human DPSCs *in vitro*. HDACi application led to a down-regulation of HDAC-3 and an increase in calcific nodule formation and associated mineralisation-marker gene expression, demonstrating the ability of HDACis to stimulate odondoblast-like differentiation *in vitro* (Jin et al., 2013). In what is currently the only *in vivo* study examining the effect of HDACis on dental pulp, the developmental effect of systemic TSA on dental tissues was investigated by injection into the tails of pregnant mice. Histological analysis revealed that the volume of dentine deposited was thicker and the number of odontoblasts in the area was higher than in the control group (Jin et al., 2013). Other studies have confirmed the promotion of mineralisation by low concentrations of VPA on DPSCs, which was accompanied by an increase in osteopontin (*OSP*) and bone sialoprotein (*BSP*) expression (Paino et al., 2014). Interestingly, the expression of osteocalcin (*OC*), a late stage marker of mineralisation, was diminished, indicating that VPA may not induce terminal differentiation. The expression levels of the genes were concomitant with inhibition of another class 1 HDAC, HDAC-2 (Paino et al., 2014). More recently, from a mechanistic viewpoint, SAHA was found to promote mineralisation and cell migration in rat DPSCs by inducing the expression of matrix metalloproteinase 13 (MMP-13). Importantly, cell proliferation was not compromised when low concentrations of SAHA were applied (Duncan et al., 2016b).

To improve the clinical relevance of the research, the interaction between HDACi and dentine matrix was investigated in order to mimic the interface between dental restorative materials and pulp engineering scaffolds at the periphery of the pulp exposure or in the root canal system (Duncan et al., 2017). The contact between materials and dentine has been shown to release bioactive dentine-matrix-components (DMCs), which promote repair events (Graham et al., 2006). The HDACi-DMC extracts contained a range of growth factors (GFs) previously identified as being released from dentine by endodontic irrigants and dental materials (Finkelman et al., 1990; Cassidy et al., 1997; Roberts-Clark and Smith, 2000; Duque et al., 2006; Graham et al., 2006; Tomson et al., 2007), as well as other novel GFs (Duncan et al., 2017). Notably, the HDACis extracted a range of GFs from dentine, less efficiently than the well-characterised extractant EDTA for certain GFs (e.g., TGF-β-1), but more effectively for others (e.g., GDF-15, BDNF), while in comparison each HDACi exhibited a different extraction profile. This work demonstrated a novel potential additional mechanism for HDACi action on reparative and regenerative dentine-pulp events in addition to established direct cellular epigenetic actions.

Over the last 10 years, isoform-specific HDAC have been developed in order to potentially reduce off-target effects and increase specificity compared with the pan-HDACis currently in use (Balasubramanian et al., 2009). Recently, it was shown that low concentrations of LMK-235, which preferentially inhibits HDAC-4 and HDAC-5, promoted odontoblast-like cell differentiation in human DPSCs without compromising cell proliferation. Furthermore, the mRNA transcript levels of mineralisation-associated genes, such as *DSPP* and alkaline phosphatase (*ALP*), were found to be elevated (Liu et al., 2018). This study lends support to the use of target-specific HDACis in stimulating odontoblast differentiation, thus introducing another opportunity for potential therapeutic application; however, in caution the role of each individual HDAC within dentine-pulp development and repair is yet to be fully elucidated.

Given the destructive nature of current treatment for pulp disease such as RCT, alternative treatment modalities using HDACis represent an attractive opportunity to harness the natural regenerative ability of the pulp. Future research should focus on the *in vivo* effects of HDACis, and investigate the potential benefit of applying HDACis to damaged pulp in order to stimulate adjacent progenitor cells to differentiate and participate in the reparative process. Furthermore, the efficacy of pan-HDACis and of target-specific HDACis should be compared, as this could potentially broaden the range of HDACis available for further therapeutic development.

### Non-coding RNAs and the Dental Pulp: Inflammation and Repair Studies

Non-coding RNAs have been shown to play a pivotal role in governing inflammation and the cellular response to damage, for example with the expression of lincRNA-Cox2 being induced by the TLR2 ligand Pam_3_CSK_4_. Furthermore, lincRNA-Cox2 has been shown to participate in both the activation and repression of other genes involved in immune response (Carpenter et al., 2013). Several miRNAs have also been implicated in inflammatory processes, such as the JAK-STAT signalling pathway, deregulation of which results in a host of immune-deficiency disorders. Within the JAK-STAT pathway, *JAK2* is targeted by miR-135a, while *STAT1* is targeted by miR-145 (Gregersen et al., 2010; Wu et al., 2012). miRNAs are also involved in the regulation of the NF-κB and MAPK pathways, both of which are major modulators of the immune response (Chew et al., 2018).

Many reparative processes are also regulated, at least in part, by miRNAs, for example with skeletal muscle regeneration after injury reliant on an intricate interaction of miRNA-regulated processes (Crist et al., 2012). MicroRNAs have also been shown to regulate each subsequent step of myogenesis, including the early differentiation of satellite cells, proliferation of myoblasts and the terminal differentiation into muscle cells (Crist et al., 2012; Liu et al., 2013; Gonçalves and Armand, 2017).

#### Dental Studies: Inflammation and Repair

**FIGURE 2:**
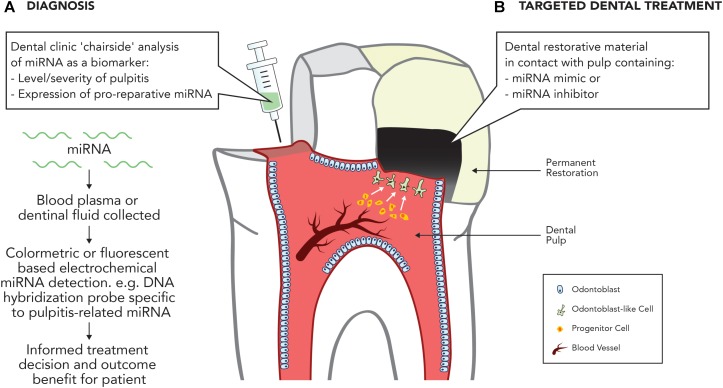
Potential applications of miRNA in treating the inflamed pulp. **(A)** Diagnosis. Development of dental ‘chairside’ analytic techniques to reliably identify inflammatory associated mRNA in pulpal blood. **(B)** Targeted therapy. Use of miRNA inhibitors or mimics to stimulate dental pulpal repair processes.

As current methods of accurately diagnosing reversible and irreversible pulpitis are crude and empirical, attention has turned to the discovery of blood borne molecular markers, which can accurately diagnose the inflammatory state of the dental pulp (Rechenberg et al., 2016). The discovery that several ncRNAs have been found to be differentially expressed in inflamed dental pulp compared with normal dental pulp highlights an opportunity to assist in this area. A recent analysis of lncRNA expression profiles found 752 lncRNAs to be significantly differentially expressed in inflamed pulp when compared with healthy pulp, with the majority being proposed to be involved in the regulation of the immune and inflammatory responses (Huang and Chen, 2018). Notably, miRNA expression is also altered during pulpitis, with 36 miRNAs significantly differentially expressed compared with uninflamed tissue (Zhong et al., 2012), including the upregulation of miR-150 and miR-584, both involved in the immune response, and miR-766, which participates in the cellular response to temperature stress. Downregulated miRNAs included miR-148 and miR-152, both negative regulators of the innate response as a well as members of the miR-181 family, including miR-181a, which regulates *IL6* (Pichiorri et al., 2008), miR-181b, which regulates motif chemokine ligand 8 (*CCL8*) (Dave and Khalili, 2010), miR-181c, which regulates *IL2* (Xue et al., 2011), and miR-181d, which regulates matrix metalloproteinase 9 (MMP-9) expression (Wang B. et al., 2010). Given the association between the deregulation of miRNAs and inflammatory diseases in other tissues, it is likely that miRNA deregulation is central to the regulation of dental pulp inflammation, which provides an opportunity for the development of miRNA-based pulpal blood diagnostics to accurately discriminate the severity of dental pulp disease (**Figure 2**).

The potential to develop ncRNA-based therapeutics for dental pulp disease has also recently become a topic of interest. Investigations into the role of ncRNAs in dentine formation and pulp mineralisation processes have revealed a number of ncRNAs associated with the regulation of odontoblast differentiation. Recently, lncRNAs have been demonstrated to participate in odontoblast-like cell differentiation of human DPSCs, with the lncRNA DANCR suppressing this differentiation process by inhibiting the wnt/β-catenin pathway (Chen et al., 2016), while miR-34a is differentially expressed in dental papillae cells throughout various stages of tooth development (Wan et al., 2012). The potential pro-mineralisation role of miR-34a is supported in osteogenesis studies, which highlight that an overexpression of miR-34a in human adipose-derived SCs (hASCs) results in a mineralisation phenotype, characterised by increased alkaline phosphatase activity and the expression of osteogenesis-associated genes (Fan et al., 2016). High-throughput microarray analysis showed altered miRNA expression profiles in DPSCs undergoing odontogenic differentiation, with 12 miRNAs upregulated and 10 downregulated. Further comparative analysis revealed that the target genes of these miRNAs were also involved in osteogenic differentiation (Gong et al., 2012). Of particular interest was miR-135b, which is significantly downregulated during pulp cell mineralisation. Given the similarities between the processes of odontogenesis and osteogenesis, and the important role of miR-135b during osteogenesis (Schaap-Oziemlak et al., 2010), a possible role for miR-135b in regulating the mineralisation of differentiating DPSCs was suggested (Gong et al., 2012). A recent study focusing on miR-135b revealed its ability to inhibit the differentiation of DPSCs via the regulation of *SMAD5* and *SMAD4* (Song et al., 2017). SMADs are involved in the bone morphogenic protein (BMP) signalling pathway, which is crucial for tooth development and dentinogenesis (Li et al., 2015). In other studies, the downregulation of miR-143-5p promoted differentiation of DPSCs into odontoblast-like cells by stimulating the expression of runt-related transcription factor 2 (*RUNX2*) (Zhan et al., 2018), while miR-675 facilitated the odontogenic differentiation of DPCs by inhibiting DNMT3B-mediated methylation of distal-less 3 (DLX3) (Zeng et al., 2018).

Recent developments in this area offer significant potential within dentistry for the development of ncRNA-based diagnostic and therapeutic strategies targeting identified miRNAs. Future research should focus on further developing our mechanistic understanding of miRNA and lncRNA expression during pulpal inflammation and repair processes and crucially investigating the *in vivo* potential of ncRNA-based therapies as tools to diagnose disease or target repair (**Figure 2**).

### Clinical and Scientific Challenges: Epigenetic-Modifying Agents in Endodontics

The therapeutic use of epigenetic-modulating agents within restorative dentistry is an exciting, but relatively recent development (Duncan et al., 2011). As a result there are a number scientific challenges and regulatory obstacles hindering clinical progress.

#### Off-Target Effects

Despite promising evidence supporting the potential use of miRNAs as therapeutic agents, a significant translational challenge relates to their lack of specificity. Although the ability of a single miRNA to silence multiple target genes is a seemingly attractive quality for the treatment of inflammation, which is characterised by the overexpression of numerous pro-inflammatory genes, this method of gene silencing will likely also silence anti-inflammatory genes (Li and Rana, 2014). It is therefore critical that prior to any proposed clinical application in teeth, a comprehensive analysis of targets specific to the miRNA of interest be carried out. Potential efforts to minimise off-target effects could include the use of miRNA masks, which bind to the miRNA-binding site in the 3′ UTR of the target mRNA. In this way, the miRNA is prevented from silencing the target mRNA, but remains free to silence non-target genes; however, this mechanism requires a comprehensive understanding of the miRNA target gene profile.

Off-target effects are also a concern with the use of HDACis, with the majority of inhibitors currently in development being pan-HDACis, designed to inhibit HDACs from classes I, II, and IV with limited or no specificity. As a result, therapeutic application of HDACis will result in a generalised non-targeted increase in histone acetylation. Furthermore, isoform-specific HDACis have proven difficult to develop, owing to the high level of HDAC sequence conservation (Benedetti et al., 2015). A potential solution is the use of a drug cocktails, which have been suggested to improve selectivity (Lehár et al., 2009). Recently, studies have shown that low doses of the HDACi, SAHA, in combination with curcumin, could improve therapeutic selectivity in the treatment of Alzheimer’s disease, providing support for the further development of a combinatorial SAHA-based drug approaches (Meng et al., 2014).

#### Delivery

A significant challenge in the use of topically applied epigenetic-modifying agents is the efficient and controlled delivery of the drug to the pulp. Although endogenous miRNAs employ several methods of protection from endonuclease-mediated digestion (Mi et al., 2013), isolated and applied ‘naked’ miRNA is unstable in circulation and quickly degraded by endonucleases (Raemdonck et al., 2008). Efforts to extend their lifespan have focused on chemical modifications to the miRNA backbone, including replacement of the phosphodiester group with phosphorothioate (Crooke et al., 1996), or substitution of the ribose 2′OH group with a 2′-O-methyl group, a 2′-O-methoxyethyl group or a 2′-fluoro group (Lam et al., 2015). Alternatively, viral vectors such as adenoviruses, adeno-associated viruses (AAVs), retroviruses and lentiviruses could offer a drug delivery solution by transfection approaches which alter local miRNA expression. Although possessing a risk of toxicity and immunogenicity, pre-clinical trials investigating the treatment of hepatocellular carcinoma with miR-26a expressed by an AAV have shown efficiency and promise, with the drug inhibiting cancer cell proliferation and simulating apoptosis (Kota et al., 2009). Recently, within a dental context, a lentivirus vector was used *in vitro* to successfully deliver miR-424 and anti-miR-424 into human DPCs to investigate their differentiation into the vascular lineage (Liu et al., 2014).

As the principle concern with viral vectors is the high risk of insertional mutagenesis and immune response, these techniques are often preferred as laboratory, rather than clinical, delivery tools. As a result, the development of non-viral vectors has been prioritised; with lipid-based delivery systems (lipoplexes) being the most commonly used non-viral delivery system. Several *in vitro* studies support the use of lipoplexes in delivering miRNA-based drugs to target cells and tissues (Ling et al., 2011; Xie et al., 2011; Zhang et al., 2011; Hara et al., 2013) as well as liposomal delivery of HDACi (Urbinati et al., 2010) for the treatment of breast cancer (Pipalia et al., 2011). Although non-viral vectors reduce the risk of insertional mutagenesis they can also be problematic *in vivo* due to their toxicity and poor transfection efficacy (Chen et al., 2015).

Another less toxic alternative is the use of nanoparticles (NPs) to deliver epigenetic-modifying agents. Inorganic nanoparticles, composed of materials such as gold, carbon and silica have the potential to be developed as non-toxic delivery systems; however, like non-viral vectors they are limited by low transfection efficiency (Tivnan et al., 2012; Ghosh et al., 2013; Jones et al., 2013; Cheng et al., 2016; Liu et al., 2017). Polymer-based nanoparticles have also been thoroughly investigated, with polymers such as Poly(lactic-*co*-glycolic acid) (PLGA) and Polyethylenimine (PEI) being incorporated into nanoparticle delivery systems (Vasir and Labhasetwar, 2007; Höbel and Aigner, 2013). Physical properties of polymer-based NPs such as molecular weight and hydrophobicity can be easily controlled, allowing the release of the active constituents to be regulated. Nanogels have also demonstrated promising qualities as carriers of epigenetic drugs, with 5-aza-2′-deoxycytidine being delivered to three cancer cell types in a nanogel delivery system; this was shown to maintain DNMT1 depletion and prolong cell-cycle arrest (Vijayaraghavalu and Labhasetwar, 2013). Within endodontics these NPs could be integrated into a dental restorative material (filling) or regenerative scaffold (**Figure 1**). This would create further interactions between the chemical constituents of the material, which would need to be functionally analysed.

Although there are a wide variety of potential delivery systems currently undergoing investigation, further research is needed within dentistry to develop a system that is capable of safe, controlled and efficient delivery, while minimising off-target effects, immunogenicity and toxicity.

#### Side Effects

Given the critical role of epigenetic modulation in the control of cell development and differentiation, there is significant concern surrounding the possibility of neoplastic transformation and tumorigenesis when developing epigenetic-based therapies to promote repair. The severe and often lethal effects observed in HDAC or DNMT-deleted mice serve as a reminder of the potential ramifications of inhibiting these broadly acting agents (Li et al., 1992; Okano et al., 1999; Haberland et al., 2009).

The side effects and off site targets of epigenetic therapeutics have been investigated in metastatic rhabdomyosarcoma (RMS) cells with high concentrations of the HDACi, TSA, and the DNMTi, 5-aza-2′-deoxycytidine, discovered to promote tumour metastasis by increasing the expression of Ezrin, a pro-metastatic protein (Yu et al., 2010). Similarly, although treatment of human breast cancer cells MCF-7 and ZR-75-1 with 5-aza-2′-deoxycytidine was shown to diminish cell proliferation and induce tumour suppressor genes, pro-metastatic genes such as plasminogen activator, urokinase (*PLAU*), which encodes urokinase (uPA), and transforming growth factor-beta 1 (*TGFβ1*) were induced via methylation of their promoters (Ateeq et al., 2008). These studies highlight the importance of a better understanding of the targets of epigenetic-modulating agents, and the need to improve the specificity of such broadly acting agents. Fortunately, within dentistry HDACis could be used in significantly lower concentrations than those reported in cancer studies (Duncan et al., 2013) and also have the potential for a topical rather than systemic application directly to the wound site, which will also reduce the chance of stimulating undesired side effects.

#### Clinical Approval

Despite the evidence supporting the use of epigenetic agents as diagnostic biomarkers or targeted therapies, there remains a significant regulatory obstacle in obtaining approval for clinical use. A 2011 study demonstrated 2136 miRNA-related patents had been established between 2000 and 2010 (van Rooij et al., 2011); however, despite this intense research and development, there are currently no Food and Drug Administration (FDA)-approved miRNA-based therapeutics. This highlights the obstacles associated with miRNA-based drugs, namely toxicity, low transfection efficacy and inefficient delivery.

The lack of approved epigenetic-based drugs can also be attributed to the lengthy process of attaining regulatory approval, with FDA approval in the United States taking an average of 12 years from the initial concept to approval for prescription (Van Norman, 2016). In contrast, the same process for medical devices (which include dental filling materials) has been reported to take an average of 3–7 years (Fargen et al., 2013). As a result, dental material development has been prioritised over medicinal product development for reasons of cost and expediency. Notably, most medicinal products do not survive beyond the approval process, with only approximately 1 in 1000 drugs being approved for clinical trials following preclinical testing (Van Norman, 2016), and only 13.8% of those graduated to clinical trials eventually being FDA-approved in the United States (Wong et al., 2018). This is perhaps the principal obstacle to the clinical development of next-generation targeted epigenetic-based therapies in the context of dentistry and specifically, regenerative endodontics. According to a recent report, dental devices in the United States currently take an average of 218 days from submission to the FDA to achieving clearance for clinical use (Emergo, 2017). However, upon introduction of an epigenetic-modifying agent, the product is classified as a drug rather than a device, and thus the approval process can be expected to take considerably longer and cost significantly more in the product development stage. These costs have a ‘knock-on’ an effect to the patient as they have to be incorporated into the product price which often in dentistry is borne by the patient rather than a third party insurance company.

#### The Need for Further Research

The use of epigenetic-modifying agents as therapeutic tools within the tooth is an exciting and novel approach. At present, although offering promise, the scope, risks and limits of epigenetic therapy are not fully understood. Given the broadly acting nature of many epigenetic-modifying agents, recent focus has been placed on improving specificity in order to decrease the risk of unwanted side-effects. Perhaps the most promising method involves combination therapy, which includes the combination of epigenetic-modulating agents with each other, cytotoxic agents and/or immunotherapeutic agents (Lehár et al., 2009; Ahuja et al., 2016). For example, the combination of HDACis with DNMTis has been under investigation as a potential treatment for acute myeloid leukaemia (AML), and has shown potential for further therapeutic development (Chen et al., 2010). More recently, the combination of several miRNAs with IL-27 has been investigated for treatment of inflammation in rheumatoid arthritis, with miR-29b, miR-21 and miR-20b delivering particularly promising results (Neto and Figueiredo, 2017).

The cellular effects of epigenetic-modulating agents vary widely between tissues and throughout cell differentiation. This, along with the interactions of these factors with each other and with their targets, generates a complex cell differentiation stage-dependent network which has not been fully elucidated. This is particularly evident for miRNAs, of which there is a rapidly growing number being identified in humans. It is therefore imperative that further research is carried out in order to fully understand the scope of miRNAs and their interactions with each other and with their targets before further therapeutic or diagnostic development.

### Current and Future Research Focus

#### Diagnostics

Further basic and translational research is required to continue development of epigenetic-based pulpal diagnostics. From a dental perspective, an accurate assessment of pulp vitality should ideally be performed at the chairside, with the patient benefitting immediately from the information relayed from the test; however, this concept is not currently employed in endodontics. Conversely, a diagnostic chairside assay for periodontal disease is currently available, which detects the level of matrix metalloproteinase 8 (MMP-8) in the gingival crevicular fluid (GCF) sampled from the gingival sulcus. The flow of GCF increases during periodontitis and its collection from the gingival sulcus is a minimally invasive procedure, involving a strip of philtre paper being gently placed between the gingival tissue and the tooth (Mäntylä et al., 2003). In contrast, the development of pulpal diagnostics has proven to be more difficult. There has been debate over whether blood is the most effective body fluid to sample (Zehnder et al., 2014) as well as concern that entering the pulp chamber will decrease the prognosis of VPT (Bjørndal et al., 2010).

Therefore, fluids such as GCF and dentinal fluid have been proposed as alternatives. GCF, although easily accessible, is problematic as tissue inflammation is a non-specific reflection of innate immunity (Hahn and Liewehr, 2007), and therefore the sample will inevitably include markers of periodontal or gingival inflammation. One possible solution to this is to ensure that periodontal tissues are healthy (Rechenberg et al., 2016); however, this is not always practical. In addition, this diagnostic method relies on an assumption that inflammatory biomarkers from the pulp will reach the gingival sulcus, a concept which has not yet been unequivocally demonstrated (Rechenberg et al., 2016). Analyses carried out on dentinal fluid samples have made use of polyvinylidene difluoride (PVDF) membranes applied to exposed dentine. MMP-9 was collected from some teeth affected with pulpitis and thus identified as a possible diagnostic biomarker; however, low yields of the protein resulted in inconsistent recovery of MMP-9 in a significant number of teeth clearly affected with pulpitis (Zehnder et al., 2011). Subsequent investigations determined that large-pore cellulose membranes were more successful in collection of potential biomarkers, with MMP-2 being highlighted as a potential diagnostic biomarker (Zehnder et al., 2014). At present, it is acknowledged that further research is needed to bring dentinal fluid-based diagnostics to the clinic.

Current studies have focused on the use of proteins as diagnostic biomarkers. Notably, miRNAs pose a distinct advantage due to their increased stability in circulation, making them a more promising biomarker for use in pulpal blood analyses (Mi et al., 2013). Indeed, further research could investigate electrochemically the analysis of miRNA biomarkers, or panels of proteins and RNAs in dentinal fluid and GCF (**Figure 2**). The recent expansion of knowledge on miRNA biomarkers has introduced a number of opportunities for a more accurate diagnosis of dental pulp disease.

#### Targeted Dental Biomaterials

Research to develop epigenetic-based therapeutic solutions for dental pulp disease has currently primarily been performed *in vitro*. Only one *in vivo* study on the effect of HDACis on dental pulp has been reported, which demonstrated promising results, supporting the need for further *in vivo* experimentation (Jin et al., 2013). Future *in vivo* research is required not only to translate these outcomes, but also to further our basic applied understanding of epigenetic mechanisms in the dental pulp and their potential focus as therapeutic targets.

In common with other topically applied drugs currently under investigation, a significant obstacle in the application of epigenetic-based therapeutics remains in their safe, controlled delivery to target tissues. Many of these drugs are rapidly degraded by host enzymes, thus necessitating the development of a suitable vehicle to protect the drug while delivering it to the target cell. Although several delivery systems are currently under investigation, there are drawbacks associated with each (Cramer et al., 2015). Recent concepts employing nanoparticles have shown promise due to their ease of synthesis and modification and their potential to be incorporated into a dental restorative material or a regenerative tissue engineering scaffold targeted at pulpal regeneration processes (**Figure 1**). This is an exciting area of research within regenerative endodontics and will unquestionably be a focus of future activity.

## Conclusion

An improved understanding of the potential therapeutic role of epigenetic-modulating agents in the regulation of dental pulp cells is emerging, which offers opportunities for the development of novel diagnostic and dental restorative biomaterials targeted at epigenetic processes. Recent research has primarily focused on the use of HDACis to simulate repair processes and promote mineralisation during development and repair. The next phase of HDACi research should focus on *in vivo* testing of the topical application of an HDACi-doped biomaterial as well as the material science aspects of integration of the inhibitor into a biological-based dental restorative biomaterial for restoration of the exposed pulp. As well as this exciting research, there is considerable potential for translational research investigating other epigenetic-modulating agents, notably ncRNAs. Further research on the role of ncRNAs, in particular miRNAs, in cellular repair and inflammatory processes, as well as the processes governing DPC differentiation, could lead to the development of ncRNA-based diagnostic and therapeutic tools for use in restorative dentistry. These tools would ideally replace the inaccurate and crude diagnostic methods currently used in restorative dentistry to assess the inflammatory state of the dental pulp, with the potential patient-benefit of avoiding the need for invasive procedures such as RCT. Potentially, miRNA could be isolated from an inflamed dental pulp sample and chairside diagnostic tests employed in order to identify previously confirmed miRNA biomarkers, which would allow accurate diagnosis and the appropriate ‘guided’ treatment to be determined. This would eliminate much of the clinical ‘guess work’ currently employed in dental practise. Furthermore, miRNA-based therapeutic dental materials could be applied to the exposed pulp to stimulate the differentiation of DPSCs into odontoblast-like cells, thus promoting dental pulp reparative processes. Research into these ncRNA approaches is relatively nascent, and so it will inevitably take some time before they can be implemented clinically. Unquestionably, several obstacles exist in the development of novel epigenetic based therapies in restorative dentistry principally in the form of creating a controlled/efficient delivery model, controlling potential off-target effects, minimising side-effects and, perhaps the most significant barrier, regulatory approval. However, given the prevalence of dental pulp disease, and the costly, invasive procedures employed in order to treat it, it is imperative that new minimally invasive solutions using epigenetic therapeutics are developed.

## Author Contributions

MK searched the literature, wrote, and edited the manuscript. AS provided guidance and edited the manuscript. PC planned, provided guidance, and edited the manuscript. HD planned, provided guidance, wrote sections, and edited the manuscript and figures.

## Conflict of Interest Statement

The authors declare that the research was conducted in the absence of any commercial or financial relationships that could be construed as a potential conflict of interest.
